# The Utility of Artificial Neural Networks for the Non-Invasive Prediction of Metabolic Syndrome Based on Personal Characteristics

**DOI:** 10.3390/ijerph17249288

**Published:** 2020-12-11

**Authors:** Feng-Hsu Wang, Chih-Ming Lin

**Affiliations:** 1 Department of Computer Science and Information Engineering, Ming Chuan University, Taoyuan 333, Taiwan; fhwang@mail.mcu.edu.tw; 2 Department of Healthcare Information and Management, Ming Chuan University, Taoyuan 333, Taiwan

**Keywords:** metabolic syndrome, artificial neural network, lifestyle factors, socioeconomic status

## Abstract

This study investigated the diagnostic accuracy of using an artificial neural network (ANN) for the prediction of metabolic syndrome (MetS) based on socioeconomic status and lifestyle factors. The data of 27,415 subjects who went through examinations and answered questionnaires during three stages from 2006 to 2014 at a health institute in Taiwan were collected and analyzed. The repeated measurements over time were set as predictive factors and used to train and test an ANN for MetS prediction. Among the subjects, 18.3%, 24.6%, and 30.1% were diagnosed with MetS during the respective three stages. ANN analysis applied with an over-sampling technique performed with an area under the curve (AUC) of up to 0.93 based on different models. The over-sampling technique helped improve prediction performance in terms of sensitivity and F_2_ measures. The results indicated that waist circumference, socioeconomic status (SES), and lifestyle factors can be utilized in a non-invasive screening tool to assist health workers in making primary care decisions when MetS is suspected. By predicting the occurrence of MetS, individuals or healthcare professionals can then develop preventive strategies in time, thus enhancing the effectiveness of health promotion.

## 1. Introduction

A number of cardiovascular risk factors (e.g., dyslipidemia, hypertension, and hyperglycemia) characterize metabolic syndrome (MetS) and result in various health problems such as cardiovascular diseases (CVD), type 2 diabetes, and fatty liver for the individuals affected by it, while also placing substantial burdens on healthcare systems; thus, early intervention is needed. In the worldwide adult population, the prevalence of MetS is estimated to be 20–25% [1]. A systematic study reported that approximately 20% or more of the adults in countries in the Asia-Pacific region suffer from MetS, with a secular increase in prevalence [2]. The potential factors underlying MetS are widely known to consist of particular demographic characteristics, socioeconomic status (SES) factors, and lifestyle behaviors. The assessment of such risk factors thus constitutes the initial step in the primary prevention of the condition and also guides associated treatment strategies since such assessment allows the strength of the preventive measures applied to be tailored to patient’s level of risk [3]. In the last decade, artificial intelligence techniques have increasingly been utilized in the processing of electronic health data, enabling such utilization to play an increasingly important role in clinical decision making. Artificial neural network (ANN) models have already been used to predict cardiovascular admission [4], coronary heart disease [5,6], and cardiovascular autonomic dysfunction [7]. Such models have also been used in previous studies to predict MetS [8,9], although the models used in those studies still required either blood testing or blood pressure measurement to be performed. At the same time, health promotion interventions cannot typically be applied to undiagnosed patients who ignore their own physical health due to a lack of biochemical or biophysical examinations. As such, a non-invasive screening tool could potentially offer considerable cost-benefit advantages in terms of large-scale screening or individual management, especially for those who rarely receive medical check-ups.

In the modified National Cholesterol Education Program – Adult Treatment Panel III, (NCEP/ATP) III criteria, there was a significant rise in the prevalence of MetS in Taiwan (from 14% to 26%) over a period of 10–15 years [10]. Yang et al. conducted a longitudinal study with a Taiwanese health screening population and suggested that the incidence of MetS was increased among women with lower education levels as they became older, and that, relatedly, SES showed a greater influence on women than on men [11]. As a chronic condition, MetS requires a patient afflicted with it not only to receive medical treatment but also to engage in long-term efforts to improve or avoid risk factors in his or her life course. A recent Taiwanese study found that there are associations between the incidence rates of MetS and CVD and alterations to the SES and long-term lifestyle behaviors of individuals. Relatedly, as the dynamic relationships between health-related outcomes and relevant factors are more clearly elucidated over time, people can be better assisted in taking more effective disease prevention measures [12]. Generally, physicians primarily rely on laboratory data to diagnose MetS. In this study, however, we sought to develop an algorithm to help physicians or others predict the occurrence of MetS over time given changes in SES factors or lifestyle factors.

## 2. Materials and Methods

### 2.1. Data Source

This study collected and analyzed data from the Major Health Screening Center in Taiwan. The center is a private, membership-oriented institute comprising four clinics, located in different parts of the country, at which the center’s roughly 0.6 million members receive periodic health examinations. In this study, each member included took part in a check-up program in which they were offered a discounted examination fee for returning repeatedly over multiple years to undergo the examination. The results of these examinations were collected in a database called the Major Longitudinal Health-check-up-based Population Database (MJLPD) that academic researchers can access upon request. Owing to the usage of the data therein, entailing various ethical issues, the protocol of this study was evaluated and agreed to by the Research Ethics Committee, National Taiwan University (NTU-REC 201612ES009), and the Major Health Screening Center.

### 2.2. Study Sample

Longitudinal data were collected for participants aged ≥20 years who received a minimum of one standard health screening at the Center during each of three three-year periods (specifically, 2006–2008, 2009–2011, and 2012–2014). All the potential participants who were found to have a cardiovascular disease of any type in the first stage were excluded from the study. Ultimately, a total of 27,415 subjects met the inclusion criteria for analysis. Some of the subjects underwent multiple screenings within one or more of the three-year periods, and for those subjects, we used the final examination within the given period for our analysis. As such, during the overall nine-year study period, for each participant, three questionnaire and examination measurements were collected, with the intervals between any two examinations ranging from one year to three years. In addition, there was a serious imbalance in the dataset, as the developed models were tested using the original dataset, resulting in imbalanced models. At the same time, a dataset was derived using an over-sampling technique, resulting in balanced models.

### 2.3. Response Variables

In the present study, MetS was defined in accordance with the modified ATP III criteria [13] and the official criteria promulgated by the National Health Promotion Administration of Taiwan. A diagnosis of MetS was made if the given participant met 3 or more of the following conditions: fasting glucose ≥100 mg/dL (5.55 mmol/L) or use of antidiabetic medication; fasting triglycerides ≥150 mg/dL; systolic blood pressure (BP) ≥130 mmHg, diastolic BP ≥85 mmHg, or use of antihypertensive medication; waist circumference (WC) ≥ 90 cm in men and ≥80 cm in women; high-density lipoprotein cholesterol <40 mg/dL in men and <50 mg/dL in women.

### 2.4. Explanatory Variables

During screening, each of the study participants completed a self-administered questionnaire regarding their sociodemographic characteristics and lifestyle habits. Aside from providing information indicating a participant’s sex and age, the data provided covered four aspects of SES and nine lifestyle habits well documented by previous studies to constitute risk factors for MetS. Due to its being easy to measure, WC (in centimeters) was also included in the model used in this study. Furthermore, medicinal treatments such as treatments consisting of antihyperglycemic, antihyperlipidemic, or antihypertensive agents were used to identify those participants who had MetS. Regarding SES, individual educational attainment, annual income, occupation, and marital status were classified into three levels. Some lifestyle habits that can be estimated in a quantitative manner in terms of frequency, such as smoking, drinking alcohol, sweetened beverage consumption, physical activity (PA), and sleep time, were categorized into three to five levels. Other lifestyle habits, including betel nut chewing, maintaining a vegetarian diet, and taking nutritional supplements, were categorized in a dichotomous manner. The variables were associated with MetS, as was reported by a previous study [12], and were all included in the current study.

### 2.5. Artificial Neural Network Models

The ANN utilized in this study consisted of a standard feedforward back-propagation ANN with three hidden layers. The multilayer perceptron (MLP) neural network adopted in this study used the RMSprop optimizer and the binary cross-entropy loss function. A 10-fold cross-validation test of the classification models built with the MLP neural network was conducted in the present study. To test the effects of the length of the predictor data on the model’s prediction performance, we built predictive models that used data from one stage, two consecutive stages, and three consecutive stages. In the one-stage models, we used stage 1, stage 2, and stage 3 predictor data to predict the target variable (i.e., MetS) in stages 1, 2, and 3, respectively. In the two-stage models, we used stages 1–2 and stages 2–3 predictors to predict MetS in stages 2 and 3, respectively. Finally, in the three-stage models, we used stage 1–2–3 predictors to predict MetS in stage 3.

In addition, as there was a serious imbalance problem in the dataset, the developed models were tested on the original dataset, resulting in imbalanced models, and on a dataset derived using an over-sampling technique, resulting in balanced models. Networks handling inputs of longer stages require larger hidden layers, with (200, 400, 400), (400, 800, 800), and (600, 1000, 1000) for one-stage, two-stage, and three-stage predictions, respectively. Furthermore, in the two-stage and three-stage networks, a dropout-layer and batch-normalization layer were used to enhance the generality of the networks’ prediction capability. 

In this study, the required number of hidden layer neurons was determined by trial and error, with the number ultimately chosen based on the goal of producing a predictive network with the most accurate estimation. In order to obtain the connection weights, the back-propagation learning algorithm, which utilizes the generalized delta learning rule, was first used to train the network. The training process is an iterative one in which the ANN computes outputs from the input datasets. These outputs are then compared to the desired outputs, and adjustments are made to the connection weights based on the errors in the outputs. At the same time, the development of an over-fitting ANN model was avoided through the test of a validation dataset with a split of 80/20, which is a separated dataset from the training datasets. The training dataset was run using the binary cross-entropy loss function, and with different settings of epochs and batch sizes. Our models were trained on a machine with the following specifications: 1× Tesla T4 graphics processing unit (GPU), compute capability 3. 7, 2496 compute unified device architecture (CUDA), an integrated technology introduced by NVIDIA cores, 12 GB GDDR5 Video RAM, 1× single core hyper threaded Xeon Processors @2.3 Ghz central processing unit (CPU), ~12.6 GB available RAM, and 312 GB available disk space. The models took about half an hour to complete the training tasks.

Furthermore, as the ratio of positive cases to negatives cases was highly imbalanced, different weights for positive samples were used for the different network models. Specifically, 50 epochs, a batch size of 100, and a weight ratio of 15:1 were used for the one-stage network prediction model; 1000 epochs, a batch size of 500, and a weight ratio of 10:1 were used for the two-stage network prediction model; and 1000 epochs, a batch size of 500, and a weight ratio of 10:1 were used for the three-stage network prediction model. The calculation of a probability value of was performed for each participant based on the derived ANN models, and the validation set was used to assess the performance in each of the developed prediction models. In the end, 10 iterations of a receiver operating characteristic curve analysis were run on the randomized test datasets in order to obtain the average AUC of receiver operating characteristic (ROC).

### 2.6. Model Evaluation

Both the calibration and discrimination of the models were measured. In this study, discrimination referred to how effectively a model could distinguish between the participants with and without MetS. For each model, the discriminatory power was analyzed according to a ROC curve and AUC, with the ROC curves used in this study being determined by plotting the false positive fraction against the true positives. For each cutoff score, the specificity (that is, the odds of a negative result given that the individual does not have MetS) and sensitivity (that is, the odds of a positive result given that the individual does have MetS) were calculated, with the cutoff score yielding the greatest sum of the sensitivity and specificity values being considered optimal. 

Due to the nature of the problem, we were more interested in the models’ ability to detect the positive cases (i.e., MetS). To this end, we used F_2_ measurements, which assign a heavier weight to recall relative to precision and consist of sums calculated by assigning a weight to sensitivity that is two times heavier relative to specificity, to estimate the accuracy of the prediction model. Meanwhile, calibration referred, in this study, to the ability of a model to make accurate predictions over the entire range. For each prediction model, the overall accuracy (which consisted of the ratio of the sum of the number of true negative and true positive results to the overall sample size) also was calculated and compared. The Hosmer–Lemeshow (HL) test was used to compute the calibration of the models, with the HL statistic providing a single summary measure of calibration resulting from a comparison of the observed and estimated prevalence rates of MetS, grouped according to the estimated prevalence. The HL statistic has a χ2 distribution, with the degrees of freedom being less than the number of groups by two.

## 3. Results

Table 1 and Table 2 present the data for the lifestyle habits, sociodemographic factors, and biochemical and physical examination items over the three study stages. MetS prevalence rates in men and women during the first stage were 26.9% and 9.7%, respectively. There was an increase in the overall MetS prevalence observed during the subsequent stages. The MetS prevalence rate for those with waist obesity increased from 67.0% at the first measurement to 80.6% at the last measurement. On the other hand, the rates of MetS prevalence differed less among those with normal and abnormal high-density lipoprotein levels, although the prevalence did increase among those with abnormal levels over the three stages.

**Figure 1:**
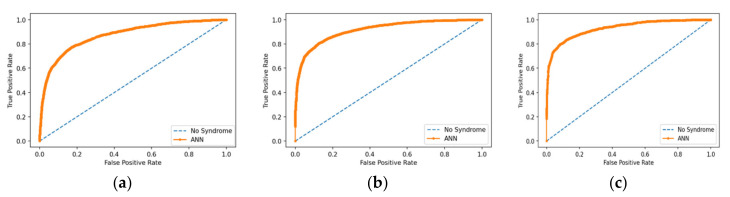
(**a**) AUC/receiver operating characteristic (ROC) curve of the balanced one-stage model; (**b**) AUC/ROC curve of the balanced two-stage model; (**c**) AUC/ROC curve of the balanced three-stage model.

Exploratory and validation sets were applied to the ANN, and three ANN models were developed to provide predictions based on one-stage, two-stage, and three-stage information. Three validation sets were randomly chosen from the data source, with 20% of the overall dataset chosen for each of these test sets. The AUC values were 0.892, 0.916, and 0.930 for the one-stage, two-stage, and the three-stage balanced ANN models, respectively, while the AUCs were 0.880, 0.885, and 0.888 for the one-stage, two-stage, and the three-stage imbalanced ANN models, respectively (Table 3). When the respective optimal cutoff points (0.4) were applied to the validation sets, the specificity and sensitivity were 82.3% and 80.7%, respectively, for the one-stage balanced ANN model; 86.7% and 80.4%, respectively, for the two-stage balanced ANN model; and 85.8% and 84.0%, respectively, for the three-stage balanced ANN model. When comparing between models, the oversampling technique helped to improve prediction performance by about 20% in terms of sensitivity with respect to the prediction performance of the critical class. The F_2_ value of the three-stage balanced ANN model was about 0.85. Meanwhile, the HL statistic values for all of the prediction models using ANN analysis were <15.0, results which indicated that the prediction models all showed good fit. Finally, the AUC/ROC curves of the balanced models are presented in Figure 1a–c.

## 4. Discussion

This study was conducted to apply ANN analyses to a dataset obtained from a large-scale longitudinal study in order to develop prediction models for MetS. The study’s key finding was that the developed prediction models are of high value in terms of their capacity to predict MetS in the clinical population. In our study, the mean AUC was 0.892 for the developed ANN models in the first stage (baseline), and the mean AUCs were 0.916 and 0.930 for two-stage and three-stage ANN models, respectively. Additionally, the sensitivity and specificity rates of the balanced models were >80%. This indicates that more than 80% of the MetS cases and non-MetS cases were successfully predicted by the balanced ANN models. These models, based on the large-scale dataset, were, thus, good-fit models (HL statistics <15.0). The average accuracy of the optimal predictive models could be up to 0.85. In comparing among the models, the over-sampling technique helped improve the prediction performance in terms of sensitivity and F_2_ measures. This is consistent with the results of Zhou and Liu, who found that over-sampling was helpful for improved prediction of the critical classes [14].

WC is a component of MetS and can be measured easily without any anthropometric device. According to the HANDLS study, for the prediction of MetS among adults, WC is the most powerful tool. Nonetheless, the appropriate index for predicting MetS and its accuracy may depend, to some degree, on gender, age, and ethnicity [15]. Yu et al. considered WC to be superior to weight-to-height ratio (WHR) and body mass index (BMI) in predicting MetS in men, while considering WHR to be superior to WC and BMI for the prediction of MetS in women [16]. A Chinese prospective cohort study reported that WC had a large AUC for the prediction of adult MetS after making adjustments for age, drinking, smoking, physical activity, and education level [17]. Using WC as one of the input variables, our study suggests that ANN tools developed with a time sequence design can provide good capacity for the prediction of MetS, irrespective of gender. 

Traditionally, linear combinations of variables are used in the LR model, such that the model is not effective at modeling grossly nonlinear complex interactions [18]. ANNs, meanwhile, constitute flexible nonlinear systems, and as such, they may be more suitable than LR-based models for the prediction of outcomes when the associations between the variables are nonlinear, complex, and multidimensional, as is the case for the relationships among variables in complex biological systems [19]. ANNs can be applied to the modeling of complex or unclear relationships, though it should also be recognized that good performance may be caused by an over-fitting model [20]. Prior knowledge of the underlying data is still required, and the current study used features that have been explored in previous research [12], which may make the results more reliable.

In a past study, Hirose et al. predicted the 6-year incidence rate of MetS by using a multiple logistic regression (MLR) analysis based on clinical factors in combination with an ANN system [9]. Applying a fuzzy neural network with a bioinformatics approach in subsequent study, Ushida et al. collected a sample of health examination data and searched for complex risk characteristics that are associated with MetS [21]. Nonetheless, these previous approaches both required the use of an invasive process to collect specimens (e.g., blood specimens) to determine biochemical or biophysical parameters. Instead of taking such inconvenient biochemical measurements, Lin et al. collected psychiatric patients’ demographic and anthropometric (i.e., BMI, blood pressure, and WC) data to predict the probability of MetS for patients treated with antipsychotics by means of an ANN and MLR. They found that both the ANN and MLR had high accuracy (88.3% vs. 83.6%, respectively) [8]. In addition to applying neural networks, two recent studies explored if applying the MetS standard definition to specific population groups by using decision tree tools is appropriate [22,23]. However, most of the aforementioned studies used a cross-sectional design, meaning that the cause–effect association needs to be further investigated. 

In recent years, various web-based healthy lifestyle programs have been developed and contributed to the reduction in MetS components [24,25]. A capable MetS prediction tool which does not require blood drawing or anthropometric measurements could potentially provide convenient and timely individual performance measurements for people with or without MetS taking part in such web-based health programs. Questionnaires regarding SES factors and lifestyle behaviors can be utilized as non-invasive screening tools that can assist health workers in making primary care decisions when MetS is suspected. By avoiding the need for data from clinical laboratory examinations, our models can provide inexpensive, non-invasive, and easy methods for identifying MetS that are not only feasible for use in assisting physicians with evaluating the health status of patients without blood draws but also for self-monitoring one’s own MetS condition when not making clinical visits.

Here are the strengths of the present study. First, it was possible, due to the use of a large sample, to explore a greater number of potential factors in the analysis while also ensuring that the amount of variance was limited, particularly with regard to the stratified sample data. Second, the time sequence design of the study made it possible to observe the changes in the outcomes and relevant factors over time in a simultaneous manner, which, in turn, allowed them to be analyzed for temporal associations between the various risk factors and outcomes that had not been as fully investigated in previous studies. Since the data sample size is quite large and a longitudinal method that we designed was used, we believe that the data can represent the real population and reduce the potential target leakage effect. Third, with respect to the artificial neural network, the over-fitting problem was addressed using validated training and by evaluating the models using test data. The potential problems posed by outliers can be treated as noise which can be tolerated by the computation models of the neural networks. The increasing prediction performance of the neural models over the sequential data demonstrates the potential of using non-invasive lifestyle factors in predicting MetS conditions.

Nonetheless, the approach of the current study had a major limitation. As members of the membership-oriented medical facility that collected the study data used, the participants could have been more health conscious or received better medical services over time than the general population. Relatedly, the participants may have had more opportunities than members of the general population to alter their adverse lifestyle habits, such as by quitting smoking, lowering their sweet beverage intake, or exercising more. They may also have been treated with or prescribed various medicines as disease interventions during their several clinic visits. In short, the effects of using the selected institution could limit the generalizability of our model to different populations. Further analyses with data from other sources are needed to ensure better generalizability.

## 5. Conclusions

In the present study, ANN models for the prediction of MetS in a clinical population were developed by using a longitudinal dataset. The developed models effectively utilized WC, SES, and lifestyle behavior data in order to serve as non-invasive screening tools to guide health workers or individuals in health decision making once MetS is suspected. By predicting the occurrence of MetS, individuals or healthcare professionals can then develop preventive strategies in time, thus enhancing the effectiveness of health promotion. However, the models’ predictive performance among different populations still needs to be investigated, such that further clarification of the external validity of the ANN models can be provided. Going forward, variable importance analysis could be conducted to identify the most prominent and influential variables and their potential theoretical and practical implications.

## Figures and Tables

**Table 1:** Metabolic syndrome (MetS)-related explanatory variables across the three study stages.

Characteristics	MetS in 2006–2008	MetS in 2009–2011	MetS in 2012–2014
	*n* (%)	*n* (%)	*n* (%)
Sex			
Male	3699 (26.9)	4822 (35.1)	5689 (41.4)
Female	1323 (9.7)	1924 (14.1)	2553 (18.7)
Age (yrs)			
30–39	1672 (12.2)	1675 (15.9)	1454 (18.7)
40–64	3004 (23.3)	4444 (28.4)	5825 (32.6)
≥65	346 (41.7)	627 (50.9)	963 (54.4)
Marital status			
Unmarried	797 (13.2)	900 (18.2)	1006 (23.8)
Married	3779 (19.9)	5197 (26.1)	6387 (31.4)
Divorced	135 (17.8)	178 (20.8)	245 (27.1)
Education (yrs)			
<12	854 (32.3)	1083 (41.8)	1252 (48.9)
12–15	2106 (17.6)	2820 (24.3)	3371 (30.0)
≥16	1941 (15.8)	2670 (21.1)	3290 (26.1)
Income (NTD/yr)			
<400,000	660 (19.8)	825 (27.0)	1045 (35.1)
400,000–799,999	1138 (16.5)	1397 (22.3)	1575 (27.5)
800,000–1,200,000	1333 (17.8)	1774 (23.4)	2054 (28.3)
>1,200,000	1466 (19.6)	2123 (25.6)	2731 (30.3)
Occupation			
Unemployed	827 (18.4)	1200 (26.4)	1573 (33.6)
Managed	645 (22.8)	859 (28.3)	1111 (35.1)
Non-managed	3115 (17.2)	4545 (22.9)	4734 (27.6)
Smoke (pack/day)			
None	3447 (17.8)	4755 (24.0)	6026 (29.6)
Ever	295 (19.6)	465 (25.4)	674 (31.1)
Exposed to passive smoke	172 (17.1)	201 (26.7)	239 (30.3)
≤1	698 (19.9)	781 (25.8)	911 (32.0)
>1	171 (20.5)	180 (26.2)	195 (29.2)
Alcohol (cup/day)			
None	3508 (16.2)	4707 (22.1)	6256 (27.7)
Ever	106 (25.2)	148 (39.4)	194 (42.0)
1	452 (24.6)	611 (33.1)	722 (37.1)
2	395 (31.3)	482 (38.2)	554 (44.4)
≥3	202 (34.8)	210 (38.4)	297 (49.9)
Sweetened beverage (cup/wk)			
None	1521 (18.6)	2967 (26.3)	4071 (31.2)
1–3	1552 (18.6)	1929 (23.9)	2161 (28.5)
4–6	809 (17.9)	757 (23.1)	779 (29.0)
≥7	1047 (17.6)	936 (22.1)	996 (28.6)
Chewing of betel nuts			
No	4387 (17.3)	5824 (23.4)	7490 (29.0)
Yes	483 (35.5)	509 (45.2)	583 (49.9)
Physical activity (h/wk)			
<1	1755 (16.0)	2483 (22.2)	2811 (28.2)
1–6	2771 (19.0)	2647 (24.7)	3525 (29.8)
≥7	290 (27.4)	573 (29.6)	743 (36.2)
Sleep (h/day)			
<6	1054 (19.7)	1650 (26.3)	2447 (32.0)
6	3363 (17.6)	3299 (24.8)	3984 (29.7)
≥7	329 (19.0)	1586 (22.4)	1852 (28.2)
Vegetarian diet			
Yes	114 (15.1)	159 (18.6)	218 (22.7)
No	4868 (18.4)	6534 (24.8)	7928 (30.0)
Vitamin C and E intake			
Yes	763 (15.8)	888 (21.1)	1065 (27.0)
No	4259 (18.9)	5858 (25.3)	7177 (30.6)
Fish oil intake			
Yes	379 (23.0)	574 (30.4)	877 (34.7)
No	4643 (18.0)	6172 (24.2)	7365 (29.6)
Related medication intake			
Yes	369 (100.0)	1205 (100.0)	1977 (100.0)
No	4653 (17.3)	5554 (21.2)	6265 (24.6)
Waist circumference in cm (sd)	86.00 (8.97)	85.59 (8.76)	85.80 (9.15)

**Table 2:** Components of metabolic syndrome (MetS) over the three study stages.

Components	MetS in 2006–2008	MetS in 2009–2011	MetS in 2012–2014
	*n* (%)	*n* (%)	*n* (%)
Waist (cm)			
<80 (F); 90 (M)	2585 (10.9)	3580 (15.3)	4243 (18.9)
Otherwise	2437 (67.0)	3166 (77.5)	3999 (80.6)
Fasting glucose (mg/dL)			
<100	979 (5.3)	1157 (7.0)	1369 (9.3)
≥100	4043 (46.0)	5589 (51.4)	6873 (54.5)
Triglyceride (mg/dL)			
<150	2335 (10.3)	3259 (14.7)	4231 (19.4)
≥150	2687 (57.6)	3487 (67.4)	4011 (72.0)
High-density lipoprotein (mg/dL)			
≥40 (M); ≥50 (F)	722 (15.0)	658 (23.8)	791 (28.3)
Otherwise	4300 (19.0)	6088 (24.7)	7451 (30.3)
Hypertension			
Normal	2092 (9.2)	3041 (13.6)	3707 (17.3)
Abnormal *	2930 (63.9)	3705 (74.1)	4535 (76.4)

* Abnormal refers to having a systolic blood pressure (BP) ≥ 130 mmHg or a diastolic BP ≥ 85 mmHg.

**Table 3:** Model predictions using an artificial neural network.

Stage Length	Model	Sensitivity	Specificity	F_2_	OA	AUC
One	Imbalanced	58.8%	95.7%	63.7%	88.5%	0.880
Two	Imbalanced	63.3%	96.7%	68.0%	89.3%	0.885
Three	Imbalanced	64.9%	95.5%	69.3%	88.1%	0.888
One	Balanced	80.7%	82.3%	81.0%	81.9%	0.892
Two	Balanced	80.4%	86.7%	81.5%	85.1%	0.916
Three	Balanced	84.0%	85.8%	84.3%	84.3%	0.930

OA: overall accuracy; AUC: area under the curve.

## References

[1] International Diabetes Federation (2006). The IDF Consensus Worldwide Definition of the Metabolic Syndrome. www.idf.org/webdata/docs/IDF_Meta_def_final.pdf.

[2] Ranasinghe P., Mathangasinghe Y., Jayawardena R., Hills A.P., Misra A. (2017). Prevalence and trends of metabolic syndrome among adults in the Asia-pacific region: A systematic review. BMC Public Health.

[3] D’Agostino R.B., Vasan R.S., Pencina M.J., Wolf P.A., Cobain M., Massaro J.M., Kannel W.B. (2008). General cardiovascular risk profile for use in primary care: The Framingham Heart Study. Circulation.

[4] Qiu H., Luo L., Su Z., Zhou L., Wang L., Chen Y. (2020). Machine learning approaches to predict peak demand days of cardiovascular admissions considering environmental exposure. BMC Med. Inform. Decis. Mak..

[5] Vallée A., Cinaud A., Blachier V., Lelong H., Safar M.E., Blacher J. (2019). Coronary heart disease diagnosis by artificial neural networks including aortic pulse wave velocity index and clinical parameters. J. Hypertens..

[6] Atkov Y.O., Gorokhova S.G., Sboev A.G., Generozov E.V., Muraseyeva E.V., Moroshkina S.Y., Cherniy N.N. (2012). Coronary heart disease diagnosis by artificial neural networks including genetic polymorphisms and clinical parameters. J. Cardiol..

[7] Liu J., Tang Z.H., Zeng F., Li Z., Zhou L. (2013). Artificial neural network models for prediction of cardiovascular autonomic dysfunction in general Chinese population. BMC Med. Inform. Decis. Mak..

[8] Lin C.C., Bai Y.M., Chen J.Y., Hwang T.J., Chen T.T., Chiu H.W., Li Y.C. (2010). Easy and low-cost identification of metabolic syndrome in patients treated with second-generation antipsychotics: Artificial neural network and logistic regression models. J. Clin. Psychiatry.

[9] Hirose H., Takayama T., Hozawa S., Hibi T., Saito I. (2011). Prediction of metabolic syndrome using artificial neural network system based on clinical data including insulin resistance index and serum adiponectin. Comput. Biol. Med..

[10] Yeh C.J., Chang H.Y., Pan W.H. (2011). Time trend of obesity, the metabolic syndrome and related dietary pattern in Taiwan: From NAHSIT 1993–1996 to NAHSIT 2005–2008. Asia. Pac. J. Clin. Nutr..

[11] Yang X., Tao Q., Sun F., Zhan S. (2012). The impact of socioeconomic status on the incidence of metabolic syndrome in a Taiwanese health screening population. Int. J. Public Health.

[12] Liao C.M., Lin C.M. (2018). Life curse effects of socioeconomic and lifestyle factors on metabolic syndrome and 10-year risk of cardiovascular disease: A longitudinal study in Taiwan adults. Int. J. Environ. Res. Public Health.

[13] Grundy S.M., Cleeman J.I., Daniels S.R., Donato K.A., Eckel R.H., Franklin B.A., Gordon D.J., Krauss R.M., Savage P.J., Smith S.C. (2005). American Heart Association; National Heart, Lung, and Blood Institute. Diagnosis and management of the metabolic syndrome. An American Heart Association/National Heart, Lung, and Blood Institute Scientific Statement. Circulation.

[14] Zhou Z.H., Liu X.Y. (2006). Training cost-sensitive neural networks with methods addressing the class imbalance problem. IEEE Trans. Knowl. Data Eng..

[15] Beydoun M.A., Kuczmarski M.T., Wang Y., Mason M.A., Evans M.K., Zonderman A.B. (2011). Receiver-operating characteristics of adiposity for metabolic syndrome: The healthy aging in neighborhoods of diversity across the life span (HANDLS) study. Public Health Nutr..

[16] Yu J., Tao Y., Tao Y., Yang S., Yu Y., Li B., Jin L. (2016). Optimal cut-off of obesity indices to predict cardiovascular disease risk factors and metabolic syndrome among adults in Northeast China. BMC Public Health.

[17] Liu L., Liu Y., Sun X., Yin Z., Li H., Deng K., Chen X., Cheng. C., Luo X., Zhang M. (2018). Identification of an obesity index for predicting metabolic syndrome by gender: The rural Chinese cohort study. BMC Endocr. Disord..

[18] Terrin N., Schmid C.H., Griffith J.L., D’Agostino R.B., Selker H.P. (2003). External validity of predictive models: A comparison of logistic regression, classification trees, and neural networks. J. Clin. Epidemiol..

[19] Greenwood D. (1991). An overview of neural networks. Behav. Sci..

[20] Ayer T., Chhatwal J., Alagoz O., Kahn C.E., Woods R.W., Burnside E.S. (2010). Informatics in radiology: Comparison of logistic regression and artificial neural network models in breast cancer risk estimation. Radiographics.

[21] Ushida Y., Kato R., Niwa K., Tanimura D., Izawa H., Yasui K., Takase T., Yoshida Y., Kawase M., Yoshida T. (2012). Combinational risk factors of metabolic syndrome identified by fuzzy neural network analysis of health-check data. BMC Med. Inform. Decis. Mak..

[22] Šabanović Š., Ljiljana M.T., Babič F., Vadovský M., Paralič J., Včev A., Holzinger. A. (2018). Metabolic syndrome in hypertensive women in the age of menopause: A case study on data from general practice electronic health records. BMC Med. Inform. Decis. Mak..

[23] Chen M.S., Chen S.H. (2019). A Data-driven assessment of the metabolic syndrome criteria for adult health management in Taiwan. Int. J. Environ. Res. Public Health.

[24] Jahangiry L., Shojaeizadeh D., Farhangi M.A., Najafi M., Mohammad K., Farhangi M.A. (2014). Modifiable lifestyle risk factors and metabolic syndrome: Opportunities for a web based preventive program. J. Res. Health Sci..

[25] Jahangiry L., Shojaeizadeh D., Farhangi M.A., Najafi M., Mohammad K., Farhangi M.A. (2015). A Interactive web-based lifestyle intervention and metabolic syndrome: Findings from the Red Ruby (a randomized controlled trial). Trials.

